# Clearing the JUNQ: the molecular machinery for sequestration, localization, and degradation of the JUNQ compartment

**DOI:** 10.3389/fmolb.2024.1427542

**Published:** 2024-08-21

**Authors:** Sarah Rolli, Chloe A. Langridge, Emily M. Sontag

**Affiliations:** Department of Biological Sciences, Marquette University, Milwaukee, WI, United States

**Keywords:** protein misfolding, proteostasis, microautophagy, piecemeal microautophagy of the nucleus, spatial sequestration

## Abstract

Cellular protein homeostasis (proteostasis) plays an essential role in regulating the folding, sequestration, and turnover of misfolded proteins via a network of chaperones and clearance factors. Previous work has shown that misfolded proteins are spatially sequestered into membrane-less compartments in the cell as part of the proteostasis process. Soluble misfolded proteins in the cytoplasm are trafficked into the juxtanuclear quality control compartment (JUNQ), and nuclear proteins are sequestered into the intranuclear quality control compartment (INQ). However, the mechanisms that control the formation, localization, and degradation of these compartments are unknown. Previously, we showed that the JUNQ migrates to the nuclear membrane adjacent to the INQ at nucleus-vacuole junctions (NVJ), and the INQ moves through the NVJ into the vacuole for clearance in an ESCRT-mediated process. Here we have investigated what mechanisms are involved in the formation, migration, and clearance of the JUNQ. We find Hsp70s Ssa1 and Ssa2 are required for JUNQ localization to the NVJ and degradation of cytoplasmic misfolded proteins. We also confirm that sequestrases Btn2 and Hsp42 sort misfolded proteins to the JUNQ or IPOD, respectively. Interestingly, proteins required for piecemeal microautophagy of the nucleus (PMN) (i.e., Nvj1, Vac8, Atg1, and Atg8) drive the formation and clearance of the JUNQ. This suggests that the JUNQ migrates to the NVJ to be cleared via microautophagy.

## Introduction

The protein homeostasis, or proteostasis, network maintains properly folded proteins in the cell (Jarosz et al., 2010; Taipale et al., 2010; Taipale et al., 2014). The mechanisms involved in the proteostasis network include protein refolding by chaperones, sequestration into membrane-less compartments, and clearance via the proteasome or autophagy (Kaganovich et al., 2008; Houck et al., 2012; Escusa-Toret et al., 2013; Sontag et al., 2023). As cells age, there is a breakdown in proteostasis mechanisms which can lead to the build-up of misfolded proteins, as seen in many neurodegenerative diseases like Alzheimer’s, Parkinson’s, and Huntington’s diseases (Ben-Zvi et al., 2009; Bar-Lavan et al., 2012; Shai et al., 2014; Shemesh et al., 2021; Cain et al., 2023). Understanding how these mechanisms work together to deal with misfolded proteins is crucial to understanding these diseases.

Chaperone machinery is responsible for recognizing misfolded or unfolded proteins. Chaperones subsequently refold the misfolded proteins into the correct conformation or target them for clearance via the proteasome or autophagy (Minoia et al., 2014; Backe et al., 2023). Many chaperones are heat shock proteins, as they were first discovered in response to heat stress (McKenzie et al., 1975; Lindquist and Craig, 1988). The main chaperone pathway for refolding proteins is the Hsp40-Hsp70-Hsp90 pathway (Velazquez and Lindquist, 1984; Borkovich et al., 1989; Chang and Lindquist, 1994; Tavaria et al., 1996). Heat shock protein Hsp40 identifies misfolded proteins and transfers them to Hsp70 for refolding (Fan et al., 2003; Walters et al., 2015; Farkas et al., 2018; Klaips et al., 2020). If the protein is severely misfolded, and Hsp70 is not able to refold it, the Hsp40-Hsp70 complex can target the misfolded protein to Hsp90, where the misfolded protein will either be refolded or targeted for degradation (Doyle et al., 2019; Bhattacharya and Picard, 2021).

Sequestration of misfolded proteins into membrane-less compartments occurs via the sorting of misfolded proteins into different compartments throughout the cell (reviewed in Sontag et al., 2017; Rolli and Sontag, 2022). Initially upon stress, cytosolic misfolded proteins form dynamic inclusions throughout the cell called Q-bodies (Escusa-Toret et al., 2013). Cytosolic misfolded proteins can be sorted into the juxtanuclear quality control compartment (JUNQ) or the insoluble protein deposit (IPOD) depending on solubility (Kaganovich et al., 2008; Escusa-Toret et al., 2013; Sontag et al., 2023) and interaction with different co-chaperones and small heat shock proteins acting as sorting factors (Specht et al., 2011; Malinovska et al., 2012; Miller et al., 2015). Nuclear misfolded proteins are sequestered into the intranuclear quality control compartment (INQ) (Gallina et al., 2015; Miller et al., 2015; Sontag et al., 2023). Recently, we showed that the JUNQ migrates to the nuclear membrane adjacent to the INQ near nuclear pores at the nucleus-vacuole junction (NVJ) (Sontag et al., 2023). Deletion of Nvj1 and Vac8, required for maintenance of the NVJ and piecemeal microautophagy of the nucleus (PMN) (Roberts et al., 2003), resulted in an increase in inclusions in both the nucleus and the cytoplasm and stabilization of protein levels.

The INQ buds into the nucleus through an Endosomal Sorting Complex Required for Transport (ESCRT)-mediated process. ESCRT machinery is responsible for vesicle budding and recruitment of protein cargo for degradation through a conveyor belt-type mechanism (Hurley and Emr, 2006; Hurley, 2015). ESCRT-I proteins, including Vps23 and Vps37, recognize cargo proteins. This recruits ESCRT-II proteins like Vps36 to start membrane nucleation. ESCRT-III (i.e., Snf7, Chm7, Vps24, etc.) sorts the cargo proteins into the vesicle, and the AAA + ATPase Vps4 then facilitates scission of the membrane and dissociation of ESCRT machinery, creating the cargo-containing multi-vesicular body (MVB) vesicle (Hurley and Emr, 2006; Hurley, 2015). Deleting Vps4 resulted in increased cells with inclusions in both the nucleus and the cytoplasm (Sontag et al., 2023). It is currently unknown if the JUNQ can also bud into the vacuole in an ESCRT-dependent manner.

Previously, we showed inhibiting vacuolar proteases showed stabilization of cytosolic misfolded proteins, suggesting cytosolic misfolded proteins are cleared by the vacuole (Sontag et al., 2023). Here, we investigated what mechanisms are required for the formation, localization, and clearance of the JUNQ by utilizing a temperature-sensitive mutant of luciferase with a nuclear export signal (NES-LuciTs). Double deletion of Hsp70s Ssa1 and Ssa2 inhibits the formation of a perinuclear JUNQ near the NVJ and promotes the formation of a static peripheral IPOD. Hsp70 deletion also stabilized NES-LuciTs protein levels. Deletion of sequestrases Btn2 and Hsp42 confirmed that NES-LuciTs is sorted to the JUNQ or IPOD in a similar way to previous studies done on other misfolded proteins (Specht et al., 2011; Malinovska et al., 2012; Miller et al., 2015). Deletion of autophagy factors Atg1 and Atg8 stabilized the levels of NES-LuciTs and increased the number of inclusions forming in the cytoplasm. NVJ proteins Nvj1 and Vac8 have been shown to be involved in the clearance of INQ and JUNQ (Sontag et al., 2023). As these proteins are known to be required for efficient PMN (Krick et al., 2008), we propose that both the JUNQ and the INQ are degraded by the vacuole at the NVJ utilizing PMN machinery.

## Methods

### Plasmids and yeast strains

Plasmids used in this manuscript are summarized in Table 1. NES-GFP-LucifersaseTs (NES-LuciTs) was expressed using a galactose-inducible promoter in pAG426 (Sontag et al., 2023). NES-DsRed-LuciferaseTs was expressed using a galactose-inducible promoter in pAG416 (Sontag et al., 2023). For *ydj1Δ* experiments, temperature sensitive Ubc9-2-mCherry was expressed using the GPD constitutive promoter in pAG416 and was a gift from Dr. Judith Frydman. Chaperone overexpression plasmids were created for this paper using the Gateway destination cloning system into GPD-EGFP-ccdB pAG413 (Alberti et al., 2007). pAG413GPD-EGFP-ccdB was a gift from Susan Lindquist (Addgene plasmid # 14310; https://n2t.net/addgene:14310; RRID:Addgene_14310). Sis1 pENTR was a gift from Dr. Anita Manogaran. Ssa1 and Hsc82 pDONR plasmids were purchased from DNASU Core Facility (Seiler et al., 2014, RRID:SCR_012185). Mutant huntingtin exon 1 protein containing an N-terminal Flag tag, the 17 N-terminal amino acids, a 97 glutamine stretch, the proline-rich domain, and a C-terminal GFP tag was expressed under a galacotse promoter on a pYES2 backbone (gift from Dr. Judith Frydman).

**TABLE 1:** Plasmids used in this manuscript.

Number	Gene of interest	Yeast marker	Bacteria marker	Backbone	Source
P-EMS-26	GPD-Ubc9-2-mCherry	URA3	Amp	pAG416	Gift from Dr. Judith Frydman
P-EMS-287	NES-GFP-Luciferase	URA3	Amp	pAG426	Sontag et al. (2023)
P-EMS-321	GPD-EGFP-ccdB	HIS3	Amp	pAG413	Gateway collection Alberti et al. (2007)
P-EMS-478	NES-DsRed-Luciferase	URA3	Amp	pAG416	Sontag et al. (2023)
P-EMS-607	Gal-Flag-N17-97QP-GFP	URA3	Amp	pYES2	Gift from Dr. Judith Frydman
P-EMS-650	pENTR Sis1		Kan	pENTR	Gift from Dr. Anita Manogaran
P-EMS-716	pDONR Ssa1		Kan	pDONR221	Horizon Seiler et al. (2014)
P-EMS-718	pDONR Hsc82		Kan	pDONR221	Horizon Seiler et al. (2014)
P-EMS-724	GPD-EGFP-Sis1	HIS3	Amp	pAG413	Created for this paper
P-EMS-725	GPD-EGFP-Ssa1	HIS3	Amp	pAG413	Created for this paper
P-EMS-726	GPD-EGFP-Hsc82	HIS3	Amp	pAG413	Created for this paper

Yeast strains used in this manuscript are summarized in Table 2. BY4741 yeast was used as a wildtype control. Deletion strains were taken from the Yeast Knockout collection (Horizon) (Wach et al., 1994; Winzeler et al., 1999; Giaever et al., 2002). Sis1 DAmP strain was taken from the Yeast DAmP collection (Horizon) (Breslow et al., 2008). The *ssa1Δssa2Δ* strain was a gift from Judith Frydman. Yeast were transformed using the One Step method (Chen et al., 1992).

**TABLE 2:** Yeast strains used in this manuscript.

Number	Background	ORF	Common name	Source
Y-EMS-1	BY4741		Wildtype background	
Y-EMS-33	BY4741	YGL180W	Atg1Δ	Horizon Wach et al. (1994), Winzeler et al. (1999), Giaever et al. (2002)
Y-EMS-36	BY4741	YBL078C	Atg8Δ	Horizon Wach et al. (1994), Winzeler et al. (1999), Giaever et al. (2002)
Y-EMS-39	BY4741	YGR142W	Btn2Δ	Horizon Wach et al. (1994), Winzeler et al. (1999), Giaever et al. (2002)
Y-EMS-62	BY4741	YDR171W	Hsp42Δ	Horizon Wach et al. (1994), Winzeler et al. (1999), Giaever et al. (2002)
Y-EMS-78	BY4741	YAL005C and YLL024C	Ssa1Δ Ssa2Δ	Gift from Dr. Judith Frydman
Y-EMS-150	BY4741	YPR189W	Vps4Δ	Horizon Wach et al. (1994), Winzeler et al. (1999), Giaever et al. (2002)
Y-EMS-154	BY4741	YBR097W	Vps15Δ	Horizon Wach et al. (1994), Winzeler et al. (1999), Giaever et al. (2002)
Y-EMS-156	BY4741	YCL008C	Vps23Δ	Horizon (Wach et al., 1994; Winzeler et al., 1999; Giaever et al., 2002)
Y-EMS-161	BY4741	YLR240W	Vps34Δ	Horizon Wach et al. (1994), Winzeler et al. (1999), Giaever et al. (2002)
Y-EMS-184	BY4741	YJL049W	Chm7Δ	Horizon Wach et al. (1994), Winzeler et al. (1999), Giaever et al. (2002)
Y-EMS-197	BY4741	YPL240C	Hsp82Δ	Horizon Wach et al. (1994), Winzeler et al. (1999), Giaever et al. (2002)
Y-EMS-201	BY4741	YAL005C	Ssa1Δ	Horizon Wach et al. (1994), Winzeler et al. (1999), Giaever et al. (2002)
Y-EMS-203	BY4741	YLL024C	Ssa2Δ	Horizon Wach et al. (1994), Winzeler et al. (1999), Giaever et al. (2002)
Y-EMS-206	BY4741	YMR186W	Hsc82Δ	Horizon Wach et al. (1994), Winzeler et al. (1999), Giaever et al. (2002)
Y-EMS-220	BY4741	YNL064C	Ydj1Δ	Horizon Wach et al. (1994), Winzeler et al. (1999), Giaever et al. (2002)
Y-EMS-223	BY4741	YNL007C	Sis1 DAmP	Horizon Breslow et al. (2008)

### Growth curve assays

Yeast cultures were grown at 30°C in selective media containing 2% raffinose to mid-log phase and diluted to an OD_600_ of 0.1 in selective media containing either 2% glucose or 2% raffinose and 2% galactose. Cultures were grown in a 96-well round-bottom plate (Falcon Cat# 351177) for 48 h at 30°C or 37°C with constant shaking. OD_600_ measurements were taken every 15 min using Molecular Devices SpectraMax Mini Microplate Reader. Sis1 DAmP samples were run separately with their own wildtype control. Wildtype controls were averaged to compare across experiments. Doubling time was calculated by non-linear regression using GraphPad Prism 10.0.1 (RRID:SCR_002798) and compared using a one-way ANOVA with Tukey’s multiple comparisons test. Area under the curve was calculated using GraphPad Prism 10.0.1 (RRID:SCR_002798) and compared using a one-way ANOVA with Tukey’s multiple comparisons test.

### Time-resolved live-cell imaging

Yeast cultures were grown as previously described (Sontag et al., 2023). Briefly, yeast were grown at 30°C in selective media containing 2% raffinose to mid-log phase and diluted to an OD_600_ of 0.2 in selective media containing 2% raffinose and 2% galactose. Cultures were grown for 1 h at 30°C to induce expression for deletion strains and for 4 h at 30°C for overexpression strains. Sis1 DAmP samples were run separately with their own wildtype control. Wildtype controls were averaged to compare across experiments. For *ydj1Δ* experiments, yeast were grown at 30°C in selective media containing 2% glucose to mid-log phase. Cells were adhered to coverslips using concanavalin A (Sigma-Aldrich Cat #C2010-250MG). Coverslips were washed with selective media containing 2% glucose and 100 μM MG132 (Sigma-Aldrich Cat# C2211-5MG) and kept in the same medium by sealing the coverslips with vacuum grease. Cells were imaged every 15 s or 1 min for 1–2 h at 37°C using Zeiss Axio Observer.Z1 7 inverted microscope (RRID:SCR_023694) equipped with X-Cite Xylis LED light source (EXCELITA Technologies), HE GFP/Cy3/DAPI shift free filter sets (Zeiss), a Plan-Apochromat 100x/1.40 oil DIC M27 objective (Zeiss), and a digital Axiocam 705 camera (Zeiss) controlled by Zen blue software (RRID:SCR_013672).

### FM4-64 vacuole labeling

Cells were grown as described for time-resolved imaging. After 3.5 h of growth in galactose medium, cells were resuspended in fresh galactose medium. FM4-64 (Invitrogen Cat #T3166) was added to a final concentration of 8 μM, and cells were incubated at 30°C for 30 min. Cells were pelleted and resuspended in selective media containing 2% glucose and 100 μM MG132 (Sigma-Aldrich Cat# C2211-5MG). Cells were heat shocked for 20 min at 37°C. Hoescht 33342 (Invitrogen Cat# H3570) was added prior to imaging to a final concentration of 8.1 μM to visualize nucleic acid. Cells were adhered to coverslips using concanavalin A (Sigma-Aldrich Cat #C2010-250MG) and washed with 100 mM HEPES pH 7.4 with 5% glucose. Images were taken using z-stacks, taking 10 stacks with a step size of 1 μm. Images were taken using Zeiss Axio Observer.Z1 7 inverted microscope equipped with X-Cite Xylis LED light source (EXCELITA Technologies), HE GFP/Cy3/DAPI shift free filter sets (Zeiss), a Plan-Apochromat 100x/1.40 oil DIC M27 objective (Zeiss), and a digital Axiocam 705 camera (Zeiss) controlled by Zen blue software (RRID:SCR_013672).

### MitoTracker mitochondria labeling

Cells were grown as described for time-resolved imaging. After 4 h of growth in galactose medium, cells were pelleted and resuspended in selective media containing 2% glucose and 100 μM MG132 (Sigma-Aldrich Cat# C2211-5MG). Cells were heat shocked for 1 h at 37°C. MitoTracker Red CMXRos (Invitrogen Cat #M7512) or Deep Red FM (Invitrogen Cat #M22426) was added to a final concentration of 8 μM for 20 min at 37°C. Hoescht 33342 (Invitrogen Cat# H3570) was added to a final concentration of 8.1 μM to visualize the nucleus. Cells were adhered to coverslips using concanavalin A (Sigma-Aldrich Cat #C2010-250MG) and washed with 100 mM HEPES pH 7.4 with 5% glucose. Images were taken as described in the FM4-64 staining section.

### Immunostaining

Cells were grown as described for time-resolved imaging. After 4 h of growth in galactose medium, cells were pelleted and resuspended in selective media containing 2% glucose and 100 μM MG132 (Sigma-Aldrich Cat# C2211-5MG). Cells were heat shocked for 2 h at 37°C. 250μL of cells were fixed with 4% paraformaldehyde for 15 min at 37°C followed by methanol fixation for 20 min at −20°C. Cells were resuspended in sorbitol buffer (1 M sorbitol, 50 mM HEPES pH 6.8, 1 mM NaN_3_) and spheroplasted with 7.5 U lyticase (Sigma-Aldrich Cat #L4025-25KU) for 75 min at 37°C. Spheroplasted cells were solubilized using 0.1% Triton X-100 for 10 min at room temperature. Nanobodies against EGFP and RFP were conjugated to Alexa Fluor 488 and Alexa Fluor 568, respectively as used previously (Sontag et al., 2023). Anti-Nsp1 antibody (EnCor Biotechnology Cat# MCA-32D6, RRID:AB_2157646) was diluted in WT buffer (1% non-fat dry milk, 0.5 mg mL^−1^ bovine serum albumin BSA, 200 mM NaCl, 50 mM HEPES–KOH (pH 7.5), 1 mM NaN_3_ and 0.1% Tween-20) and cells were incubated in antibody for 2 h at room temperature. Cells were washed with WT buffer and incubated in secondary antibody Alexa Fluor 647 goat anti-mouse IgG (Thermo Fisher Scientific Cat# A-21235, RRID:AB_2535804) for 2 h at room temperature. Cells were adhered to coverslips using poly-lysine-coated coverslips (Sigma-Aldrich Cat# P8920-100ML) and mounted in Prolong Diamond mounting media with DAPI (Thermo Fisher Cat# P36962). Images were taken as described in the FM4-64 staining section.

### Photobleaching microscopy

Cells were grown as described for time-resolved imaging. After 4 h of growth in galactose medium, cells were pelleted and resuspended in selective media containing 2% glucose and 100 μM MG132 (Sigma-Aldrich Cat# C2211-5MG). Cells were heat shocked for 2 h at 37°C. Mutant huntingtin exon 1 protein (HTT) was used as a control for IPOD formation and photobleaching (Kaganovich et al., 2008). HTT expressing cells were grown in 2% galactose, 2% raffinose for 24 h at 30°C prior to imaging. Cells were adhered to coverslips using concanavalin A (Sigma-Aldrich Cat #C2010-250MG). Photobleaching was performed on a Nikon TE2000 Perfect Focus Ti - E microscope with phase contrast, epi-fluorescence, DIC, and resonant scanning confocal system for 405, 488, 561, and 638 nm lasers (RRID:SCR_023161) controlled by Nikon NIS-Elements software (RRID:SCR_014329). Cells were imaged pre-photobleaching, half of an inclusion was bleached using the 488 nm laser for 5 s, and cells were then imaged every 2 s for 90 s post-bleaching.

### Microscopy image analysis

Images were analyzed using ImageJ (RRID:SCR_003070) and Volocity (RRID:SCR_002668). No alterations were made to the time-lapse images prior to puncta counting analyses. Background subtraction in Zen using a radius of 100. Photobleaching correction (v2.1.0 exponential fit) (Miura, 2020) and registration (descriptor-based series (2d/3d + t) nearest neighbor, fuse and display with Σ_1_ = 12.901999, Σ_2_ = 15.343149, Threshold = 0.012469863) (Preibisch et al., 2010) were performed on all time lapse data after puncta counting analyses using ImageJ. 3D reconstructions were performed in Volocity with adjustments to black and white levels to optimize visualization. Photobleaching image analysis was performed using ImageJ. Integrated density measurements using a drawn ROI were normalized to the pre-bleach image.

### SDS-PAGE with Western blotting

Yeast cultures were grown as described previously (Sontag et al., 2023). Cultures were grown at 30°C in selective media containing 2% raffinose to mid-log phase and diluted to an OD_600_ of 0.7 in selective media containing 2% raffinose and 2% galactose. Cultures were grown for 4 h at 30°C before pelleting and resuspending in selective media containing 2% glucose with or without 50 μM bortezomib (LC Laboratories Cat# B-1408) (Samant et al., 2018; Sontag et al., 2023). Cultures were shifted to 37°C for 4 h. Samples were taken every 2 h and pelleted. For *ydj1Δ* experiments, cells were grown at 30°C in selective media containing 2% glucose to mid-log phase and diluted to an OD600 of 0.7 in selective media containing 2% glucose. Cultures were grown for an hour prior to treating with 50 μM cycloheximide (Millipore Sigma Cat# 239763-1GM) and 50 μM bortezomib (LC Laboratories Cat# B-1408) and shifting to 37°C for 1 h. Samples were taken every 30 min and pelleted.

Cells were lysed in urea lysis buffer (8 M urea, 50 mM HEPES pH 7.4, Roche cOmplete protease inhibitor Cat# 11836170001) and vortexed with glass beads. 20% SDS was added to a final concentration of 4% SDS, and lysate was incubated at 65°C for 5 min. Lysate was pelleted, and protein concentration of the supernatant was measured with the bicinchoninic acid protein assay kit (Thermo Fisher Cat# 23250).

Equal amounts of total protein were resolved on 12% Tris-glycine gels and transferred to nitrocellulose (BioRad Cat# 1620112). EGFP was detected with mouse anti-GFP monoclonal antibody (Roche Cat# 11814460001, RRID:AB_390913), DsRed and mCherry were detected with Living Colors rabbit anti-DsRed polyclonal antibody (Takara Bio Cat# 632496, RRID:AB_10013483), and GAPDH was detected with mouse anti-GAPDH monoclonal antibody (Genetex Cat# GT239, RRID:AB_11174761). Secondary antibodies used were rabbit Anti-Mouse IgG (H + L), HRP Conjugate (Promega Cat# W4021 (also W402B), RRID:AB_430834) and goat Anti-Rabbit IgG (H + L), HRP Conjugate (Promega Cat# W4011 (also W401B), RRID:AB_430833). Membranes were imaged using Clarity Western ECL Substrate (Biorad Cat# 170-5,061) on GE Amersham Imager 600. NES-LuciTs band intensities were measured using ImageJ (RRID:SCR_003070) and normalized to the GAPDH control. Degradation ratios of 2h–0h and 4h–0 h were calculated for each sample and normalized to the wildtype control to allow for comparison between blots.

## Results

### Hsp40s and 70s are important for sorting of cytoplasmic misfolded proteins to compartments

**FIGURE 1:**
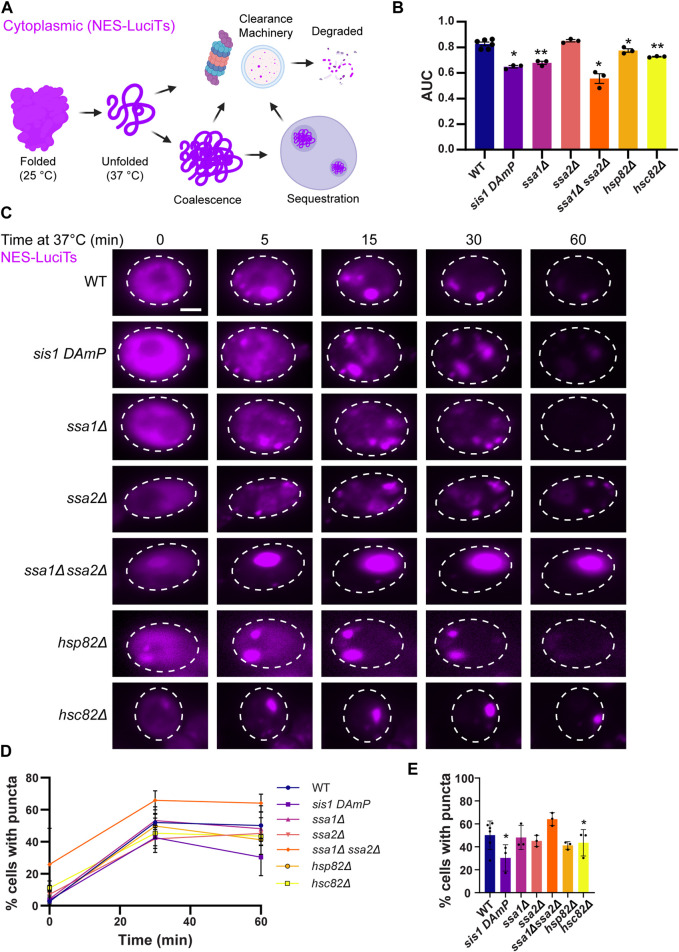
Hsp40s and 70s are important for sorting of cytoplasmic misfolded proteins to compartments. **(A)** Schematic showing a temperature sensitive mutant of Luciferase with a nuclear export signal tag (NES-LuciTs) misfolds when temperature is shifted to 37°C. We are investigating what pathways dictate the coalescence, sequestration, and clearance of NES-LuciTs. **(B)** Area under the curve (AUC) analysis of growth curves of wildtype BY4741 and chaperone deletion strains expressing NES-LuciTs at 37°C in 2% galactose and 2% raffinose selective media normalized to 2% glucose controls. Paired t-tests: *P-value<0.02. **P-value<0.001 **(C)** Time-resolved live-cell microcopy of wildtype BY4741 and chaperone deletion strains expressing NES-LuciTs at 37°C (magenta). Representative still frames at the times shown. Three replicates were performed. **(D)** Percentage of cells with puncta over time. **(E)** Percentage of cells with puncta at 60 min. Paired t-tests: *p-value<0.05. All error bars represent standard deviation.

Previous work found that nuclear and cytoplasmic misfolded proteins are cleared by the proteasome and the vacuole (Sontag et al., 2023). However, the clearance of nuclear misfolded proteins occurs faster than clearance from the cytoplasm (2 h vs. 4 h, respectively) and is more impacted by proteasome inhibition. Further, there is a slight growth defect in cells expressing NES-LuciTs at the misfolded temperature (37°C) compared to the partially folded temperature (30°C). There is no growth defect seen with NLS-LuciTs at either temperature (Sontag et al., 2023). Therefore, we investigated sorting factors and clearance mechanisms of cytoplasmic misfolded proteins, particularly those sorted into the JUNQ compartment (Figure 1A).

We began by investigating how loss of Hsp40, Hsp70, and Hsp90 affects the formation and clearance of the JUNQ. We examined the growth of yeast expressing NES-LuciTs at 37°C with depleted Hsp40 Sis1 levels by using the DAmP strain, Hsp70s Ssa1 and Ssa2, and Hps90s Hsp82 and Hsc82 deletions from the yeast deletion collection (Wach et al., 1994; Winzeler et al., 1999; Giaever et al., 2002; Breslow et al., 2008). We also investigated a double deletion strain of Ssa1 and Ssa2. Double deletion of Ssa1 and Ssa2 leads to decreased thermotolerance and altered protein quality control, even though Ssa4 is upregulated (Craig and Jacobsen, 1984; Matsumoto et al., 2005). Reducing or eliminating Sis1, Ssa1, Hsp82, Hsc82, and the double deletion of Ssa1 and Ssa2 resulted in a growth defect when NES-LuciTs was misfolded (Figure 1B). This indicates that the chaperones are playing a role in assisting proteostasis of misfolded cytoplasmic proteins. To account for any differences in growth at 37°C in the chaperone mutants themselves, we normalized the values of the area under the curve (AUC) with expression of the NES-LuciTs to the AUC of the strains grown at 37°C without the expression of NES-LuciTs. AUC measurements for the strains grown at 37°C with and without expression of NES-LuciTs are shown in Supplementary Figures S1A, B.

We then used live cell time-lapse fluorescence microscopy to determine if there were defects in sorting or clearance of misfolded NES-LuciTs in the chaperone mutant strains. While the mutant strains did still show formation and clearance of inclusions, it appeared that there was a defect in the coalescence of Q-bodies during the middle timepoints of the experiment, especially upon depletion of Sis1 and deletion of Ssa1 or Ssa2 (Figure 1C). Hsp82, Hsc82, and the Ssa1 Ssa2 double deletion each formed inclusions earlier than the WT, with small inclusions visible at time 0. Hsc82 and the Ssa1 Ssa2 double deletion strains both formed 1 large inclusion, and the inclusion in the Hsc82 deletion strain was more mobile than the one formed in the Ssa1 Ssa2 double deletion (Figure 1C). We counted the number of cells containing puncta in each of the strains and found that depleting Sis1 and deleting Hsp82 resulted in fewer cells with puncta after 60 min (Figures 1D, E). While the Ssa1 Ssa2 double deletion had more cells with puncta at all 3 time points, it was not statistically significantly higher (T0 *p* = 0.187, T30 *p* = 0.1115, T60 *p* = 0.089). Proteasome impairment did not have a major effect on the coalescence of Q-bodies into the JUNQ or on the percentage of cells with puncta (Supplementary Figures S1C–E).

Given the effect of Sis1 depletion on sorting and toxicity, we tested the other major cytosolic Hsp40 Ydj1 (Caplan and Douglas, 1991). Because Ydj1 is required for the induction of galactose-inducible promoter (Floer et al., 2008), we were not able to investigate the effect of deletion of Ydj1 on NES-LuciTs using our galactose-inducible NES-LuciTs system. We performed time-lapse experiments and clearance Western blots using a constitutively expressed temperature sensitive mutant of Ubc9 (Ubc9-2-mCherry) that showed that deletion of Ydj1 causes delayed coalescence over time, but Ydj1 deletion does not impact clearance (Supplementary Figures S2A, B), as was found previously (Escusa-Toret et al., 2013).

### Hsp70s are required for JUNQ formation

**FIGURE 2:**
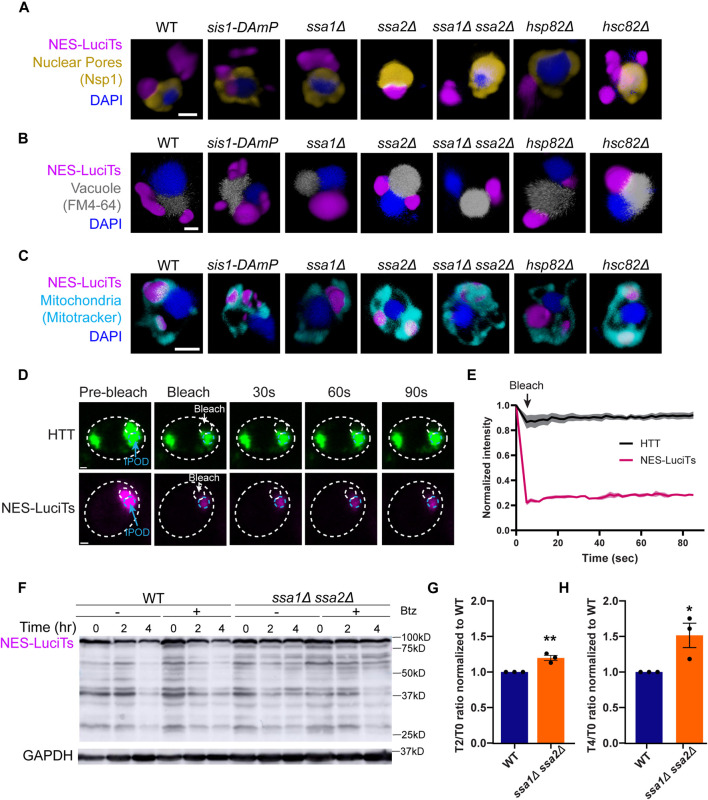
Hsp70s are required for JUNQ formation. **(A–C)** 3D reconstructions of cells expressing NES-LuciTs (magenta) created in Volocity. **(A)** Representative images of nuclear pore staining with anti-Nsp1 (gold) on fixed cells in DAPI staining (blue) after 2 h heat shock with MG132 treatment. **(B)** FM4-64 vacuole staining of live cells (gray) with Hoechst 33342 staining (blue) after 20 min heat shock with MG132 treatment. **(C)** Representative images of MitoTracker Red CMXRos staining of live cells (cyan) with Hoechst 33342 staining (blue) after 20 min heat shock with MG132 treatment. All scale bars are 1 μm. **(D, E)** Photobleaching experiments on ssa1Δssa2Δ cells expressing NES-LuciTs or BY4741 wildtype cells expressing Flag-97QP-GFP as an IPOD control. Cells were imaged pre-bleach, bleached for 5 s, and imaged every 2 s for 90 s post-bleach. **(E)** Intensity of the inclusion over time normalized to the pre-bleach intensity. **(F)** Representative Western blot of NES-LuciTs in ssa1Δssa2Δ. Samples were collected at 0, 2, and 4 h after shifting to 37°C. Cells were treated with or without 50 μM bortezomib to inhibit the proteasome. Anti-GFP antibody was used to detect NES-LuciTs (1 s exposure). Anti-GAPDH antibody was used to detect GAPDH (1 s exposure). **(G, H)** Quantification of NES-LuciTs bands. Band intensities were measured using ImageJ and normalized to GAPDH control. Degradation ratios between 2 h **(G)** or 4 h **(H)** to time 0 were calculated and normalized to wildtype to allow for comparison between blots. Unpaired *t*-test: **P-value = 0.0061. *P-value = 0.0392. Error bars represent standard error of the mean.

To confirm that the JUNQ is still formed in the chaperone mutant strains, we investigated the location of the inclusions relative to the nucleus, vacuole, and mitochondria. We found that the inclusions formed in single chaperone mutants still localize to the nuclear periphery (Figure 2A), at nucleus-vacuole junctions (Figure 2B), and inclusions from all of the strains are proximal to mitochondria (Figure 2C). This indicates that the perinuclear inclusions seen in the chaperone single deletion mutants are likely to be the JUNQ as these are all characteristics of the JUNQ shown previously (Sontag et al., 2023). This is likely due to other paralogs of Hsp70 and Hsp90 compensating for the loss of the single chaperone, allowing the JUNQ to still form, even if Q-body coalescence is slowed.

In *ssa1Δssa2Δ* cells, one inclusion forms per cell prior to heat shock which persists over time (Figure 1C) and is in the periphery of the cell (Figures 2A–C). To determine if this inclusion was static like the IPOD has been described previously (Kaganovich et al., 2008), we performed photobleaching experiments where we bleached half of the inclusion and tracked the fluorescence intensity in the other half over time. We found that the single inclusion in *ssa1Δssa2Δ* does not recover fluorescence intensity and remains static like the IPOD formed from mutant huntingtin protein (Figures 2D, E). Double deletion of Ssa1 and Ssa2 also inhibited clearance of NES-LuciTs compared to wildtype (Figures 2F–H), suggesting that Hsp70s are required for the clearance of NES-LuciTs. This occurred both with and without proteasome impairment (Figure 2F). We conclude that Ssa1 and Ssa2 play a role in JUNQ sorting, and deletion of Hsp70s promotes IPOD formation and inhibits clearance.

### Overexpression of 40, 70, and 90 did not alter sequestration

**FIGURE 3:**
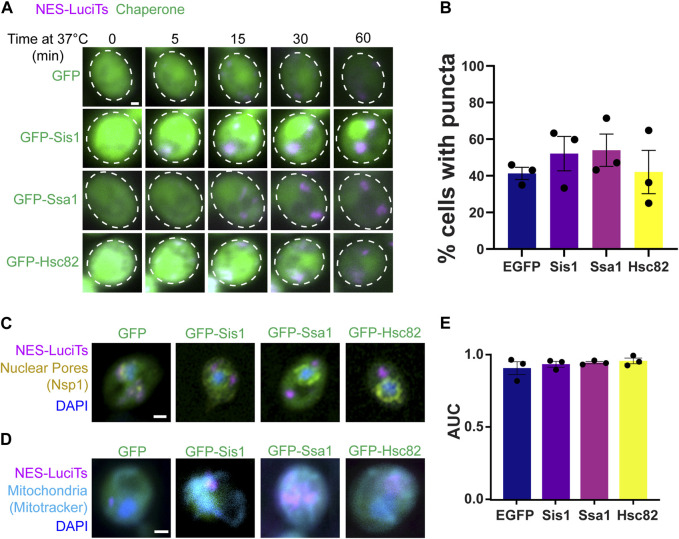
Overexpression of chaperones does not affect NES-LuciTs localization. **(A)** Time-resolved live-cell microscopy of wildtype BY4741 expressing NES-LuciTs (magenta) and overexpressing GFP-tagged chaperones or GFP alone (green). Representative still frames at the times shown. Three replicates were performed. **(B)** Percentage of cells with puncta from the time-resolved imaging at 60 min. One-way ANOVA F = 0.5495, *p*-value = 0.6625. **(C)** Representative images of nuclear pore staining with anti-Nsp1 on fixed cells in DAPI staining (blue) after 2 h heat shock with MG132 treatment. **(D)** Representative images of MitoTracker Deep Red FM staining of live cells (cyan) with Hoechst 33342 staining (blue) after 20 min heat shock with MG132 treatment. All scale bars are 1 μm. **(E)** Area under the curve (AUC) analysis of growth curves of wildtype BY4741 overexpressing EGFP, Sis1, Ssa1, or Hsc82 and expressing NES-LuciTs at 37°C in 2% galactose and 2% raffinose selective media normalized to 2% glucose controls. One-way ANOVA F = 0.7747, *p*-value = 0.5401.

Next, we wanted to determine if overexpression of Hsp40, Hsp70, or Hsp90 altered the formation or clearance of the JUNQ. We found no change in the formation of cytoplasmic inclusions when Sis1, Ssa1, or Hsc82 were overexpressed in yeast expressing NES-LuciTs at 37°C by live cell time-lapse microscopy (Figure 3A) or by counting inclusions after 60 min at 37°C (Figure 3B). The inclusions were still located at the nuclear periphery (Figure 3C) and in the proximity of mitochondria (Figure 3D). To determine if overexpression of Hsp40, Hsp70, or Hsp90 altered the toxicity of NES-LuciTs, we performed growth assays at 37°C where the LuciTs is misfolded. We saw no change in the AUC of the growth curves of yeast overexpressing chaperones at 37°C versus GFP expression alone (Figure 3E). Together, these data suggest over-expressing these chaperones individually does not change inclusion formation or clearance, likely due to the increased expression of these chaperones upon heat shock in our experiments.

### Sequestrases Btn2 and Hsp42 are involved in the sorting of misfolded proteins to different cytoplasmic PQC compartments

**FIGURE 4:**
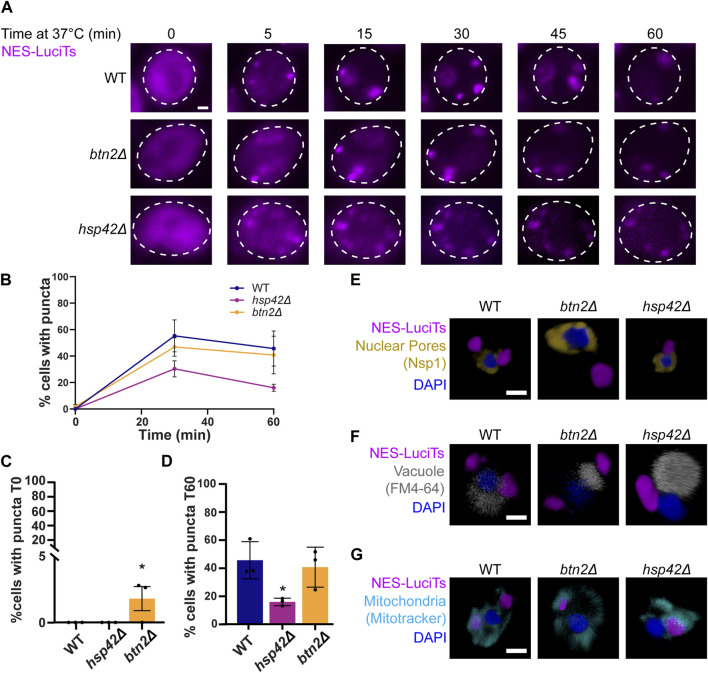
Hsp42 and Btn2 are involved in the sorting of NES-LuciTs. **(A)** Time-resolved live-cell microcopy of wildtype BY4741, *btn2Δ*, and *hsp42Δ* expressing NES-LuciTs at 37°C (magenta). Representative still frames at the times shown. Three replicates were performed. **(B)** Percentage of cells with puncta over time. **(C)** Percentage of cells with puncta at 0 min. One-way ANOVA F = 5.920, *p*-value = 0.0380. Dunnett’s multiple comparison test: *p-value = 0.0357. **(D)** Percentage of cells with puncta at 60 min. One-way ANOVA F = 6.504, *p*-value = 0.0038. Dunnett’s multiple comparison test: *p-value = 0.0323. Error bars represent standard error of the mean. **(E–G)** 3D reconstructions of cells expressing NES-LuciTs (magenta) created in Volocity. **(E)** Representative images of nuclear pore staining with anti-Nsp1 (gold) on fixed cells in DAPI staining (blue) after 2 h heat shock with MG132 treatment. **(F)** Representative images of FM4-64 vacuole staining of live cells (gray) with Hoechst 33342 staining (blue) after 20 min heat shock with MG132 treatment. **(G)** Representative images of MitoTracker Red CMXRos staining of live cells (cyan) with Hoechst 33342 staining (blue) after 20 min heat shock with MG132 treatment. All scale bars are 1 μm.

Btn2 and Hsp42 have been previously shown to sort misfolded proteins to cytoplasmic compartments and are often referred to as sequestrases (Specht et al., 2011; Malinovska et al., 2012; Miller et al., 2015). To ensure that the addition of the NES tag was not altering interactions with these sequestrases and subsequent compartmentalization, we examined NES-LuciTs sorting in yeast strains lacking either Btn2 or Hsp42. The sequestrase mutant strains formed inclusions as expected by live cell time-lapse fluorescence microscopy (Figure 4A). We found that deletion of Hsp42 reduces the percentage of cells with puncta (Figures 4B, D). Deletion of Btn2 causes there to be significantly more puncta prior to heat shock, but percentage of cells with puncta over time during heat shock is not significantly different (Figures 4C, D).

Using immunofluorescence, we confirmed that the misfolded NES-LuciTs was being sorted to different locations depending on the identity of the sequestrase present. In the *btn2Δ* strain, misfolded NES-LuciTs was sorted to the periphery of the cell (Figures 4E, F). Conversely, *hsp42Δ* yeast sorted misfolded NES-LuciTs to a perinuclear compartment at the nucleus-vacuole junction that is likely the JUNQ (Figures 4E, F). The inclusions formed in the sequestrase mutants were still wrapped in mitochondria as seen in WT and other yeast strains (Figure 4G). These data confirm previous reports for sorting cytoplasmic misfolded proteins (Specht et al., 2011; Malinovska et al., 2012) and indicates that the NES localization tag does not change interactions with the sequestrases.

### The JUNQ is cleared in the vacuole via PMN

**FIGURE 5:**
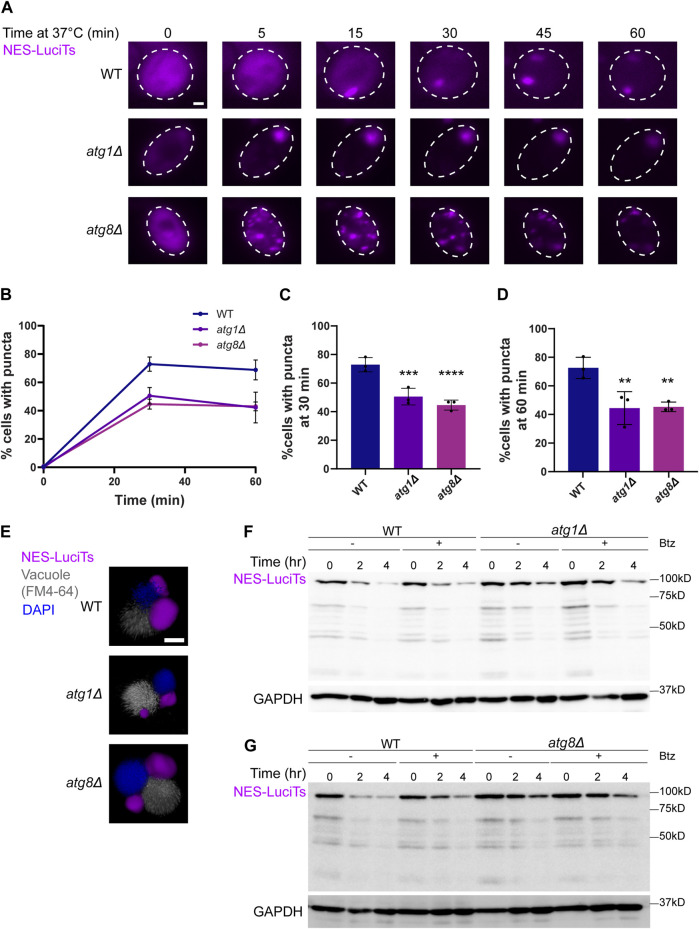
Autophagy proteins Atg1 and Atg8 are involved in the clearance of NES-Luci. **(A)** Time-resolved live-cell microcopy of wildtype BY4741, *atg1Δ*, and *atg8Δ* expressing NES-LuciTs at 37°C (magenta). Three replicates were performed. **(B)** Percentage of cells with puncta over time. **(C)** Percentage of cells with puncta at 30 min. One-way ANOVA F = 14.85, *p*-value<0.0001. Dunnett’s multiple comparison test: ***p-value = 0.0007. ****p-value<0.0001. **(D)** Percentage of cells with puncta at 60 min. One-way ANOVA F = 7.635, *p*-value = 0.0019. Dunnett’s multiple comparison test: **p-value = 0.004. Error bars represent standard error of the mean. **(E)** 3D reconstructions of cells expressing NES-LuciTs (magenta) with FM4-64 (gray) and Hoechst 33342 (blue) staining after 20 min heat shock with MG132 treatment. Created using Volocity. All scale bars are 1 μm. **(F, G)** Representative Western blots of NES-LuciTs in *atg1Δ* **(F)** and *atg8Δ* **(G)**. Samples were collected at 0, 2, and 4 h after shifting to 37°C. Cells were treated with or without 50 μM bortezomib to inhibit the proteasome. Anti-GFP antibody was used to detect NES-LuciTs (1 s exposure). Anti-GAPDH antibody was used to detect GAPDH (1 s exposure).

We then examined the involvement of the autophagy pathway in vacuolar degradation of the JUNQ as the INQ and other protein aggregates are degraded in the vacuole (Lu et al., 2014; Marshall et al., 2016; Sontag et al., 2023). We began by looking at sequestration and clearance of NES-LuciTs in deletions of core autophagy proteins Atg1 and Atg8. Atg1 is a kinase required for the formation of the phagophore assembly site and vesicle formation in autophagy (Cheong et al., 2008). Atg8 is the yeast homolog of the mammalian LC3 protein that becomes conjugated to phosphatidylethanolamine and is responsible for membrane fusion and phagophore expansion during autophagosome formation (Xie et al., 2008). Live cell time-lapse fluorescence microscopy showed that cytoplasmic inclusions still form in *atg1Δ* and *atg8Δ* yeast (Figure 5A). Deletion of Atg1 leads to more cells with a single large inclusion, while Atg8 deletion causes more puncta per cell that do not coalesce over time. These deletions led to a reduced percentage of cells with puncta at both 30 and 60 min of heat shock (Figures 5B–D).

Immunofluorescence microscopy showed that inclusions still formed at the NVJ in the *atg1Δ* and *atg8Δ* yeast cells (Figure 5E). We see that NES-LuciTs degradation is slowed in *atg1Δ* and even more *atg8Δ* yeast (Figures 5F, G). Inhibiting the proteasome with Bortezomib had no impact on the degradation of NES-LuciTs in WT, *atg1Δ*, or *atg8Δ* yeast (Figures 5F, G), supporting the conclusion that autophagy plays a bigger role in clearance of the NES-LuciTs than the proteasome.

We had previously found that there are more cells with nuclear and cytoplasmic inclusions in *nvj1Δ* and *vac8Δ* yeast and that the INQ cannot enter the vacuole without the NVJ (Sontag et al., 2023). Nvj1 and Vac8 work with the core autophagy machinery, including Atg1 and Atg8, to degrade portions of the nucleus through PMN (Krick et al., 2008). We also showed that the ESCRT-I protein required for ubiquitin-dependent protein sorting, Vps23, is involved in the budding of the INQ from the nucleus into the vacuole. The ESCRT-II/III protein Chm7 is required for the recruitment of the INQ and the JUNQ to the NVJ. Despite the roles of these ESCRT proteins in INQ clearance and INQ and JUNQ localization, there is no change in the percentage of cells with puncta in *vps23Δ* and *chm7Δ* yeast (Supplementary Figure S2C). Together, these data indicate a role for PMN in the clearance of the JUNQ as well as the INQ.

## Discussion

**FIGURE 6:**
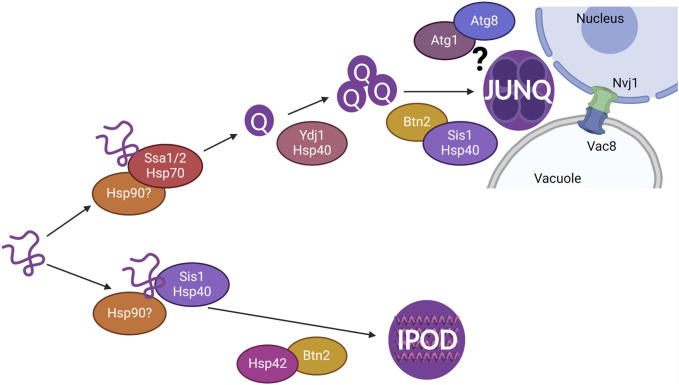
Model showing chaperones involved in the sequestration of misfolded cytoplasmic proteins resulting in the formation of Q-bodies, JUNQ, and IPOD. The JUNQ resides at the nucleus-vacuole junction (NVJ) where autophagy proteins such as Atg1 and Atg8 and NVJ proteins Nvj1 and Vac8 are likely involved in the vacuolar degradation of the JUNQ.

Misfolded proteins are sequestered into membrane-less protein quality control compartments throughout the cell. We previously showed that the INQ is likely cleared via PMN (Sontag et al., 2023). We found that the JUNQ homes in to the nucleus and they are both cleared via the vacuole (Sontag et al., 2023). Here, we investigated several mechanisms to determine what is required for the clearance of cytoplasmic proteins and how these mechanisms are involved in the formation and localization of the JUNQ (summarized in Figure 6).

Chaperones recognize misfolded proteins for refolding and are known to colocalize to the JUNQ (Malinovska et al., 2012; Escusa-Toret et al., 2013). We investigated the role of Hsp40, Hsp70, and Hsp90 in the formation, localization, and clearance of cytoplasmic NES-Luci. Reduction of Sis1 using the Sis1 DAmP strain led to a defect in the maturation of Q-bodies, shown by delayed coalescence. However, the JUNQ is still able to form, and clearance is not impacted. This is similar to the role of Ydj1 in Q-body coalescence but not clearance as previously shown (Escusa-Toret et al., 2013) and in Supplementary Figure S2. Deletion of Hsp70s Ssa1 and Ssa2 together promotes the formation of the IPOD and inhibits clearance of cytoplasmic misfolded proteins (Figure 2). This suggests that Hsp70s are needed for the sorting of misfolded proteins to the JUNQ, and the other Hsp70s Ssa3 and Ssa4 are not able to compensate for the roles of Ssa1 and Ssa2 in cytoplasmic misfolded protein sequestration. Hsp90 likely has a role in the sequestration of cytoplasmic misfolded proteins, but Hsp82 and Hsc82 are able to compensate for the other in most conditions. Overall, this suggests that Hsp70s are recognizing cytoplasmic misfolded proteins for sequestration into the JUNQ and clearance.

While deletion of both Ssa1 and Ssa2 led to the formation of an IPOD that did not recover fluorescence upon photobleaching, the inclusion formed by NES-Luci was more sensitive to the photobleaching than mutant huntingtin protein that is known to form the IPOD. This resulted in a larger drop in normalized intensity upon bleaching of the NES-Luci inclusion compared to the mHTT (Figures 2D, E). This could be due to the mutant huntingtin protein forming an amyloid containing IPOD whereas the NES-Luci forms a static IPOD but does not contain amyloid. Future studies will determine if the IPOD has different biophysical properties when it contains amyloids.

To verify we were able to assess changes in JUNQ formation and localization in our NES-Luci system, we used Hsp42 and Btn2 deletion strains to observe puncta formation over time and localization in relation to the nucleus, the vacuole, and mitochondria. The JUNQ is known to form at NVJ, and mitochondria form a cage around the JUNQ. Wildtype cells show the formation of a JUNQ and IPOD for NES-Luci. Hsp42 is required for the formation of peripheral foci, and not perinuclear foci (Specht et al., 2011). Deletion of Hsp42 caused a reduction in the peripheral foci formed by NES-Luci, with foci being predominantly perinuclear, likely the JUNQ (Figure 4). Btn2 is required for perinuclear foci formation (Malinovska et al., 2012), and we find that deletion of Btn2 causes NES-Luci to only form peripheral foci, likely the IPOD (Figure 4). NES-Luci is forming a JUNQ and an IPOD, verifying that the NES tag does not alter the sorting of misfolded proteins into the JUNQ and IPOD.

We then decided to investigate canonical autophagy proteins Atg1 and Atg8. Atg1 is a protein serine/threonine kinase that is required for the formation of vesicles in canonical autophagy (Cheong et al., 2008). Atg8 targets Atg1 to autophagosomes and is required for membrane fusion in autophagosome formation (Xie et al., 2008). Deletion of Atg1 or Atg8 caused a decrease in the clearance of NES-Luci compared to wildtype. Inhibiting the proteasome had no significant impact on the degradation of NES-LuciTs by Western blot (Figures 2F, 5F–G). This supports the conclusion that NES-Luci is being cleared via autophagy.

Degradation of NES-VHL has been shown to be slowed by proteasome inhibition (Samant et al., 2018). We also see a different time scale for clearance of NES-Luci than was seen for NES-VHL as the NES-VHL appears to be cleared in approximately half the time of NES-Luci (Samant et al., 2018; Sontag et al., 2023;Figures 2F, 5F–G). The VHL protein is constitutively misfolded in yeast as it lacks its cofactors Elongin B and C (McClellan et al., 2005). It is possible that constitutively misfolded proteins are handled differently than those that misfold upon temperature stress. We are currently investigating the role of co-translational chaperone interactions with these proteins and the difference in sequestration as a result of the co-translational binding of chaperones.

Because the Endosomal Sorting Complex Required for Transport (ESCRT) is responsible for nuclear budding of the INQ and homing of the INQ and the JUNQ (Sontag et al., 2023), as well as facilitating autophagy via formation and fusion of autophagosomes, we investigated if these ESCRT proteins identified as important for INQ clearance have a role in the degradation of cytoplasmic proteins. We find that deletion of Vps23 and Chm7 have no effect on puncta formation despite their roles in INQ budding from the nucleus and INQ-JUNQ homing, respectively (Supplementary Figure S2C).

Vps15 is a ubiquitin-binding kinase involved in targeting proteins to the vacuole (Herman et al., 1991). Vps34 and Vps15 are both components of the PI3K complex at the NVJ (Stack et al., 1993) and play a role in INQ nuclear budding into the vacuole. While it is unknown if Vps34 and Vps15 directly play a role in PMN, the PI3K complex is required for efficient PMN (Krick et al., 2008). We investigated the impact of Vps15 and Vps34 on the formation and clearance of the JUNQ, but we are not able to conclude anything from our results due to expression level issues of NES-LuciTs, likely due to their role in transcription elongation (Gaur et al., 2013). Future work will be done to investigate mutant forms of these proteins that still allow for sufficient expression of NES-LuciTs. As PMN occurs at NVJs and requires Atg1, Atg8, and the PI3K complex, it is likely that PMN is clearing not only the INQ, but the JUNQ as well.

We know that the INQ enters the vacuole via a membrane budding mechanism (Sontag et al., 2023), but it is unclear if the JUNQ enters the vacuole via a membrane bound structure or can be directly engulfed by the vacuole. It is possible that the JUNQ utilizes the same machinery as PMN but does not enter the vacuole via microautophagy mechanisms like budding. It is also possible that the JUNQ stays fused to the outside of the nuclear bud containing the INQ and is therefore pulled into the vacuole during INQ clearance. Future studies will determine the full protein requirements and mechanism for JUNQ clearance via the vacuole.

Ubiquitination has been proposed as a mechanism for moving proteins from the IPOD to the JUNQ (Kaganovich et al., 2008). It is possible that ubiquitination can alter the interactions with chaperones to control how it is sequestered and cleared. It is also possible that other forms of selective autophagy could be involved or act as compensatory mechanisms when other pathways are blocked. Cue5, the yeast homolog of TOLLIP, can mediate autophagy of ubiquitinated protein aggregates by acting as a cargo adaptor to form Atg8-coated autophagosomes (Lu et al., 2014; Marshall et al., 2016). Future studies will investigate the roles of ubiquitin and other forms of selective autophagy in the clearance of the JUNQ.

While we still have much to learn about sequestration and degradation of misfolded proteins, we find that the formation, localization, and clearance of the JUNQ is dependent on chaperones, sequestrases, and the microautophagy machinery. It is still unclear how microautophagy is clearing the JUNQ and the step in the formation and localization of the JUNQ that the chaperones are playing a role. Based on our current findings, it is likely that Hsp40s are more involved in the coalescence of Q-bodies into the JUNQ, and Hsp70s are more involved either at or within the JUNQ. The role of Hsp90 is still unclear and will require future studies using a double deletion strain with temperature-sensitive mutants of Hsp82 or Hsc82 to determine their function in JUNQ formation and clearance. Future studies will elucidate the timing of JUNQ clearance through PMN relative to the INQ. It also remains unknown if these findings are specific to model proteins such as NES-LuciTs or apply to endogenous misfolded protein substrates and disease-associated proteins as well. Understanding these differences will provide a fundamental understanding of how misfolded proteins are handled in the cell, and how that goes awry in disease.

## Data Availability

The datasets presented in this article are not readily available because there are no large datasets associated with the manuscript. Requests to access the datasets should be directed to emily.sontag@marquette.edu.
