# Supplementary figures and images for: Clearing the JUNQ: the molecular machinery for sequestration, localization, and degradation of the JUNQ compartment
Source: Front Mol Biosci. 2024 Aug 21;11:1427542. doi: 10.3389/fmolb.2024.1427542 (PMC11372896; doi:10.3389/fmolb.2024.1427542)

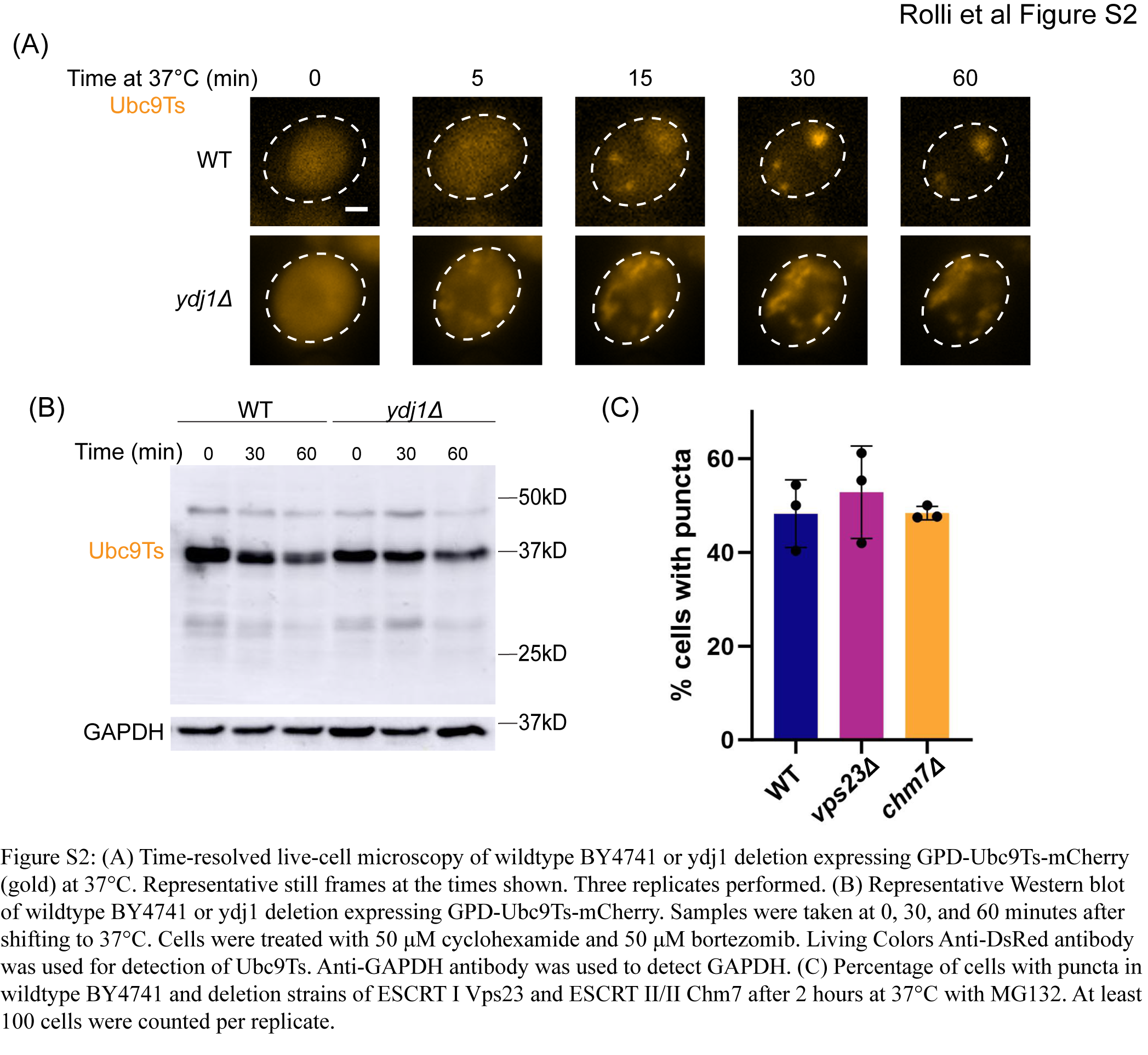

Supplement: Supplementary file 1 [file Image2.tif]

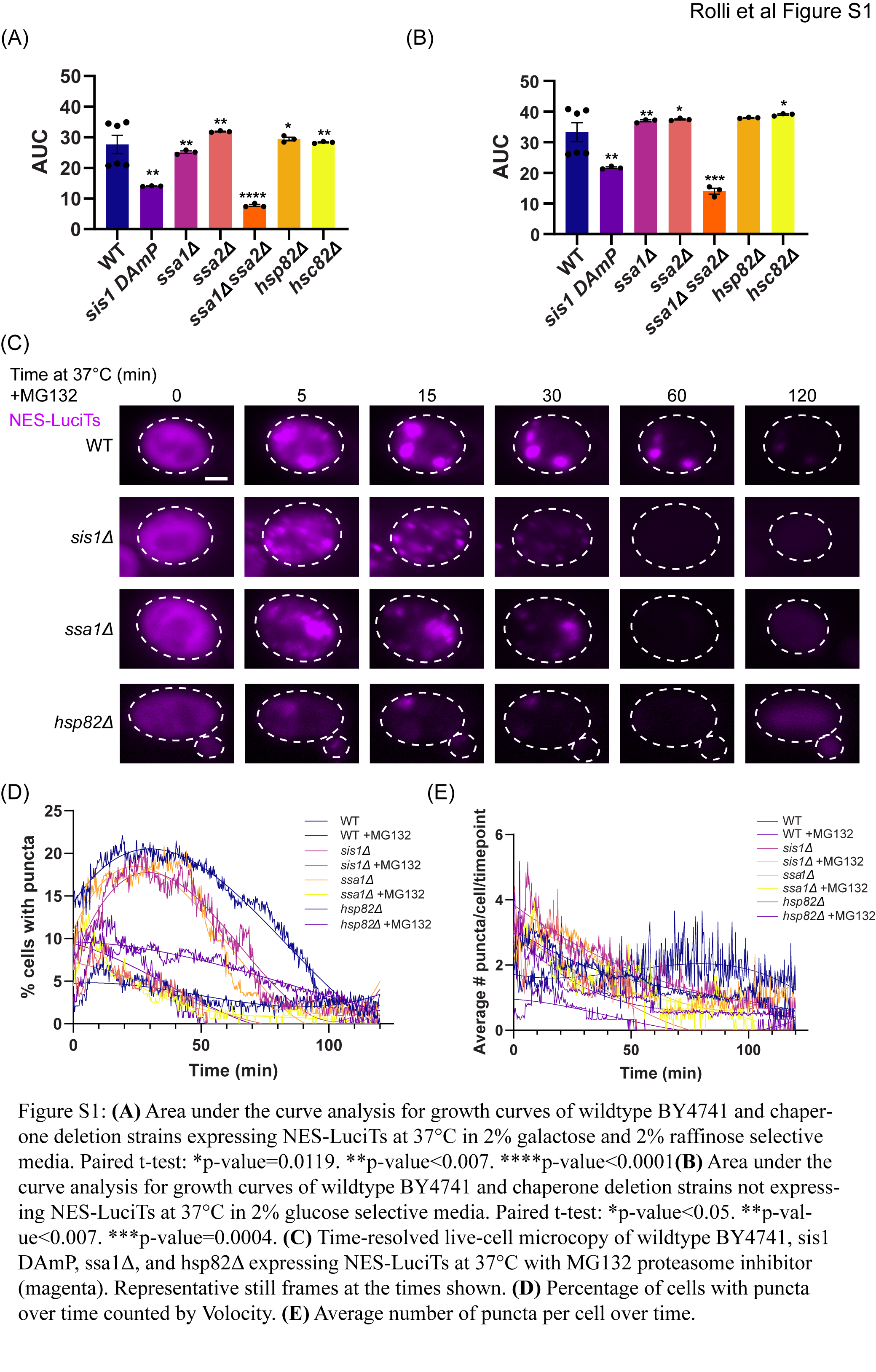

Supplement: Supplementary file 2 [file Image1.tif]
